# Tolerogenic Effect Elicited by Protein Fraction Derived From Different Formulas for Dietary Treatment of Cow’s Milk Allergy in Human Cells

**DOI:** 10.3389/fimmu.2020.604075

**Published:** 2021-02-12

**Authors:** Lorella Paparo, Gianluca Picariello, Cristina Bruno, Laura Pisapia, Valentina Canale, Antonietta Sarracino, Rita Nocerino, Laura Carucci, Linda Cosenza, Tommaso Cozzolino, Roberto Berni Canani

**Affiliations:** ^1^ Department of Translational Medical Science, University of Naples “Federico II”, Naples, Italy; ^2^ CEINGE Advanced Biotechnologies, University of Naples “Federico II”, Naples, Italy; ^3^ Institute of Food Sciences, National Research Council (CNR), Avellino, Italy; ^4^ European Laboratory for the Investigation of Food-Induced Diseases, University of Naples Federico II, Naples, Italy; ^5^ Task Force for Microbiome Studies, University of Naples Federico II, Naples, Italy

**Keywords:** immune tolerance, gut barrier, hypoallergenic formulas, soy formula, amino acid formula, extensively hydrolyzed casein formula, extensively hydrolyzed whey formula, rice formula

## Abstract

Several formulas are available for the dietary treatment of cow’s milk allergy (CMA). Clinical data suggest potentially different effect on immune tolerance elicited by these formulas. We aimed to comparatively evaluate the tolerogenic effect elicited by the protein fraction of different formulas available for the dietary treatment of CMA. Five formulas were compared: extensively hydrolyzed whey formula (EHWF), extensively hydrolyzed casein formula (EHCF), hydrolyzed rice formula (HRF), soy formula (SF), and amino acid-based formula (AAF). The formulas were reconstituted in water according to the manufacturer’s instructions and subjected to an *in vitro* infant gut simulated digestion using a sequential gastric and duodenal static model. Protein fraction was then purified and used for the experiments on non-immune and immune components of tolerance network in human enterocytes and in peripheral mononuclear blood cells (PBMCs). We assessed epithelial layer permeability and tight junction proteins (occludin and zonula occludens-1, ZO-1), mucin 5AC, IL-33, and thymic stromal lymphopoietin (TSLP) in human enterocytes. In addition, Th1/Th2 cytokine response and Tregs activation were investigated in PBMCs from IgE-mediated CMA infants. EHCF-derived protein fraction positively modulated the expression of gut barrier components (mucin 5AC, occludin and ZO-1) in human enterocytes, while SF was able to stimulate the expression of occludin only. EHWF and HRF protein fractions elicited a significant increase in TSLP production, while IL-33 release was significantly increased by HRF and SF protein fractions in human enterocytes. Only EHCF-derived protein fraction elicited an increase of the tolerogenic cytokines production (IL-10, IFN-*γ*) and of activated CD4+FoxP3+ Treg number, through *NFAT*, *AP1*, and *Nf-Kb1* pathway. The effect paralleled with an up-regulation of *FoxP3* demethylation rate. Protein fraction from all the study formulas was unable to induce Th2 cytokines production. The results suggest a different regulatory action on tolerogenic mechanisms elicited by protein fraction from different formulas commonly used for CMA management. EHCF-derived protein fraction was able to elicit tolerogenic effect through at least in part an epigenetic modulation of *FoxP3* gene. These results could explain the different clinical effects observed on immune tolerance acquisition in CMA patients and on allergy prevention in children at risk for atopy observed using EHCF.

## Introduction

Cow’s milk allergy (CMA) is an important problem worldwide with lifelong implications for health. With an estimated prevalence up to 3% it is one of the most common food allergies and one of the main causes of food-induced anaphylaxis in the pediatric age (1–6). Cow’s milk allergy derives from a breakdown of immune tolerance mechanisms leading to abnormal immune-mediated response to proteins in cow’s milk, that occurs consistently with ingestion (7). The condition imposes a significant cost to the health care systems as well as to families, and it emerged as one of the most expensive allergic diseases (8, 9). Whatever the clinical pattern of CMA, the mainstay of treatment is the elimination from the diet of cow’s milk proteins. If breastfeeding is not available, the child must be fed a special formula adapted to CMA dietary management, during infancy and later, if the disease persists. This type of formula must be adequate in terms of allergic and nutritional safety. The most used are the following: extensively hydrolyzed whey (EHWF) or casein formula (EHCF), soy formula (SF), hydrolyzed rice formula (HRF) or amino acid-based formula (AAF) (10). Special formulas emerged as the primary cost driver for CMA management (11). It has been estimated that the cost to feed a child with CMA is 2.5 to 6 times higher (10). Thus, options to accelerate the process of immune tolerance acquisition would be very welcomed by affected families and health care systems.

Special formulas are traditionally considered mostly for management, but not a cure, of the disease. Formulas available for CMA treatment differ mainly regarding the protein fraction features, such as source (cow’s milk, soy, or rice), degree and procedure of hydrolysis (12). It has been suggested that selected milk protein hydrolysates may be able not only to avoid allergic symptoms in CMA children due to the destruction of IgE epitopes, but might also have immune-modulating properties like the induction of T cell tolerance and the prevention of sensitization (7). An increasing number of data suggest a potentially different impact on immune tolerance acquisition induced by different formulas available for CMA management (3, 5, 13–19). There is also evidence that selected cow’s milk protein-based hydrolyzed infant formulas may have a long-lasting preventive effect on the development of allergy in children at risk for atopy (20–24). This evidence points out the importance to better define the potential tolerogenic effects elicited by the protein fraction from formulas commonly used for CMA treatment.

The study aimed to comparatively evaluate the tolerogenic effect elicited by the protein fraction derived from different formulas available for the dietary treatment of CMA.

## Methods

### Study Design

The study was designed as comparative blinded evaluation of the effects elicited by protein fraction from different formulas on main non-immune and immune components of the tolerance network in human enterocytes and peripheral mononuclear blood cells (PBMCs). In total, five different commercially available formulas were analyzed: EHCF, EHWF, HRF, SF, and AAF.

To simulate what happens in the infant gut lumen after oral administration, each formula was subjected to an *in vitro* simulated infant gut digestion. We assessed, in a blinded manner, gut barrier permeability, tight-junction proteins involved in the regulation of gut barrier integrity (occludin and zonula occludens-1, ZO-1), mucin 5AC (Muc5AC) protein involved in the regulation of gut barrier mucus thickness, and the epithelial cell-derived danger signal mediators, interleukin (IL)-33 and thymic stromal lymphopoietin (TSLP) in human enterocytes. Whereas, the Th1/Th2 cytokine response and Tregs activation were investigated in PBMCs from IgE-mediated CMA infants. Only after completion of the analysis, the formula identity was disclosed.

### Study Formulas’ Protein Fraction Preparation

In **Table S1** were reported the main features of the study formulas. The commercially available study formulas were reconstituted in tap water according to the manufacturer’s instructions. Protein content in the formulas was determined by measuring protein nitrogen as previously described (25). Aliquots corresponding to 500 mg of protein fraction were subjected to an *in vitro* simulated infant gut digestion using a harmonized sequential gastric and duodenal static model, as previously described (26). Simulated digestion was performed in disposable sterile plastic tubes. Compared with the adult model, the substrate-to-enzyme ratio of pepsin (gastric phase) and pancreatin (duodenal phase) was reduced eightfold and tenfold, respectively, in order to mirror those of the infant digestion process (27). Similarly, gastric phospholipids and bile salts were tenfold reduced. Formulas were incubated 2 h for both the simulated gastric and duodenal phases. Immediately after duodenal incubation, the digests were centrifuged (3,000 g, 15 min, 4°C), and the floating lipid layer was removed. The resulting protein digests were purified using C18 reversed phase pre-packed cartridges (Sep-Pak, Waters, Milford, MA, USA). Protein fractions were recovered in 70% acetonitrile/0.1% trifluoroacetic acid and finally vacuum-dried. The protein fraction purification steps were carried out with toxin-free disposable devices. To limit the bias potentially induced by LPS contamination, we assessed in each study formula-derived protein fraction the LPS content as previously described (12). To remove also traces of LPS contamination, a two-phase detergent-based (Triton X-114) extraction was also performed, as previously described (28). To evaluate possible inter-batches variability, three commercially available batches from each study formula were digested *in vitro* and analyzed by nanoflow HPLC coupled with high resolution mass spectrometry (Orbitrap technology).

The protein fractions purified from three different batches of the EHCF were identified from the merged replicate analyses using the Andromeda search engine of MaxQuant open source bioinformatic suite (version 1.6.2.10). Relative amount of peptides was inferred by signal ion count. Analysis and software-assisted identification workflows were performed as previously described (29). Peptide maps were visualized using the Peptigram web application (http://bioware.ucd.ie/peptigram/).

### Human Enterocyte Cell Lines

Caco-2 cells were obtained from the American Type Tissue Culture Collection (ATCC^®^ HTB-37, Teddington, UK). NCM460 cells were purchased from INCELL Corporation (San Antonio, TX, USA). Both cell lines were grown in Dulbecco’s modified Eagle’s medium (DMEM; Gibco, Berlin, Germany) with a high glucose concentration (4.5 g/L) and L-glutamine, supplemented with 10% fetal bovine serum (FBS, Gibco) 1% non-essential amino acids (Gibco), 1% sodium pyruvate (Gibco), 1% penicillin/streptomycin (Gibco). The cells were incubated at 37°C in a humidified atmosphere containing 5% CO2. The culture medium was changed every 2 days.

### Blood Sampling and Isolation of Peripheral Mononuclear Blood Cells

Peripheral blood samples were obtained from six IgE-mediated, challenge-proven, CMA infants. The main demographic and clinical features of the study subjects are depicted in **Table S2**. Blood samples were analyzed in an anonymized manner with the permission of the Ethics Committee of the University Federico II of Naples. Informed written consent was obtained from parents/tutors of each patient. PBMCs were isolated from heparinized peripheral blood (8 ml) by Ficoll density gradient centrifugation (Ficoll Histopaque-1077, Sigma, St. Louis, Missouri, USA). Briefly, cells were stratified on 3 ml of Ficoll and centrifuged 15 min at 2,000 rpm at room temperature. After centrifugation, the opaque interface containing mononuclear cells was carefully aspirated with a Pasteur pipette and cells were washed with 10 ml of PBS and centrifuged 10 min at 1,200 rpm at room temperature. After centrifugation, the upper layer was discarded and PBMCs (2 × 10^5^ cells/well) were cultured in duplicates in 96-well plates in 200 µl culture medium (RPMI 1640, Gibco) containing 10% FBS (Gibco), 1% non-essential amino acids (Gibco), 1% sodium pyruvate (Gibco), and 1% penicillin/streptomycin (Gibco).

### Human Enterocytes Stimulation Protocol

Caco-2 and NCM460 cells were stimulated after 15 days post-confluence cultured in six-well plates. The epithelial monolayer was stimulated with digested protein fraction derived from different formulas (EHCF, EHWF, HRF, SF, AAF) or with *β*-lactoglobulin (BLG) or bovine serum albumin (BSA), as control, at 25 µg/ml for 48 h. Afterward, the supernatants were harvested and stored at −20°C for further use. Cells with only medium were also used as negative control. BLG and BSA were purchased from Sigma (Sigma-Aldrich, Milan, Italy). To remove endotoxin from BSA and BLG, a two-phase detergent-based (Triton X-114) extraction was performed, as previously described (28). PBMCs were incubated at 37°C in a humidified atmosphere with 5% CO_2_ for 5 days, and stimulated with 25 µg/ml of digested protein fraction derived from the study formulas (EHCF, EHWF, HRF, SF, AAF) or with BLG or BSA, as control. Cells with only medium were also used as negative control. After the incubation period, culture supernatants were collected to assess Tregs and Th1 and Th2 cytokines.

### Transepithelial Electrical Resistance

To evaluate the monolayer integrity by transepithelial electrical resistance (TEER), 2 × 10^6^ Caco-2 and NCM460 cells per well were seeded on polycarbonate 6-well Transwell^®^ membranes (Corning, Life Science, Kennebunk, USA). The TEER was measured every 24 h for a total of 72 h, using an epithelial Volt-Ohm Meter (Millicel-ERS-2, Millipore, Billerica, MA, USA). The measured resistance value was multiplied by the area of the filter to obtain an absolute value of TEER, expressed as Ω cm^2^, and the TEER values were measured as follows: TEER = (measured resistance value − blank value) × single cell layer surface area (cm^2^).

### Quantitative Real-Time PCR

Total RNA was extracted with TRIzol reagent (Gibco BRL, Paisley, UK) and reverse transcribed in cDNA with a High-Capacity RNA-to-cDNA™ Kit (Life Technologies, Waltham, MA, USA) according to the manufacturer’s instructions. Complementary DNA (cDNA) was stored at −80°C until use. Quantitative real-time PCR (qRT-PCR) analysis was performed using Taqman Gene Expression Master Mix (Applied Biosystems, Vilnius, Lithuania) to evaluate the gene expression of mucin5AC (Muc5AC; Hs01365616_m1) and tight junction proteins occluding (Hs05465837_g1) and ZO-1 (Hs01551871_m1), CD137 (Hs00155512_m1), NFAT5 (Hs00232437_m1), AP1 (Hs99999141_s1), and Nf-kB1 (Hs00765730_m1). The TaqMan probes for these genes were inventoried and tested by Applied Biosystems manufacturing facility (QC). The amplification protocol was 40 cycles of 15 s of denaturation at 95°C, 60 s of annealing at 60°C, and 60 s of elongation at 60°C in a Light Cycler 7900HT (Applied Biosystems, Grand Island, NY, USA). Data were analyzed using the comparative threshold cycle method. We used the glyceraldehyde-3-phosphate dehydrogenase (GAPDH) gene to normalize the level of mRNA expression.

### Assessment of IL-33 and TSLP

The epithelial cell-derived danger signal mediators, IL-33 and TSLP, were assessed in Caco-2 cells culture media, using commercially available ELISA kits specific for human IL-33 (BioVendor Research and Diagnostic Products, Karasek, Brno, Czech Republic; detection limit of 0.2 pg/ml) and for human TSLP (Elabscience Biotechnology Inc. Wuhan, Hubei; detection limit of 18.75 pg/ml), respectively. The ELISAs were conducted according to the manufacturer’s recommendations.

### Assessment of Th1/Th2 Cytokines in PBMCs Culture Supernatant

The concentrations of IL-4 and IL-10 were measured with a Human IL4/IL10 Enzyme immunoassay kit (Boster Biological Technology, Ltd., Fremont, CA, USA). The IL-5, IFN-*γ* and IL-13 concentrations were measured using the human ELISA assay kit (BioVendor). The minimum detection concentrations were 1.5 pg/ml for IL-4, 7.8 pg/ml for IL-5, 0.5 pg/ml for IL-10, 0.99 pg/ml for IFN-*γ*, and 0.7 pg/ml for IL-13. The ELISAs were conducted according to the manufacturer’s recommendations.

### Treg Population Analysis by Flow Cytometry and Cell Sorting

Tregs were identified as CD4+/CD25+/FoxP3 positive cells by flow cytometry analysis. The staining was performed using Treg detection human kit (Miltenyi Biotech, Bergisch Gladbach, Germany) and the results analyzed by BD CANTO II flow cytometer and DIVA software (Becton-Dickinson, Franklin Lakes, New Jersey, USA). A total of 100,000 events were acquired for analysis after gating of lymphocytes based on the FSC/SSC dot plot. For sorting, labeled cells were sorted using a FACs Aria I sorter (BD Biosciences). For Treg isolation, a CD4+CD25^veryhi^ gate was used, and sorted cells were collected in media (RPMI/20% FBS), washed once, and suspended in culture media.

### DNA Methylation Analysis

DNA was extracted from sorted Tregs from stimulated PBMCs, using the DNA Extraction Kit (GE Healthcare). One microgram of extracted DNA was modified with sodium bisulfite to convert all unmethylated, but not methylated-cytosines to uracil. Bisulfite conversion was carried out using the EZ DNA Methylation Gold Kit (ZYMO Research Co., Orange, CA, USA), according to the manufacturer’s instructions. The converted DNA was stored at −70°C until used. Fully methylated and fully unmethylated DNA (Merck Millipore, Darmstadt, Germany) were used as controls for the optimization of the assay conditions and to calculate the percent of methylation (0 to 100%). The primers used for DNA methylation analysis of IFN-*γ*, IL-10, and *FoxP3* in Treg-specific-demethylation-region (TSDR) are reported elsewhere (30). High-resolution melting real-time PCR for methylation analysis was performed as described previously (31). The results of methylation analysis were verified by direct sequencing (using the Sanger method modified as follows: ddNTPs labeled with four different fluorophores) and analyzed by capillary electrophoresis (the analytical specificity and sensitivity of the test was >99%).

### Statistical Analysis

The Kolmogorov–Smirnov test was used to determine whether variables were normally distributed. Descriptive statistics were reported as means and standard deviations (SDs) for continuous variables. To evaluate the differences among continuous variables, the independent sample t-test was performed. The level of significance for all statistical tests was two-sided, p< 0.05. All data were collected in a dedicated database and analyzed by a statistician using GraphPad Prism 7 (La Jolla, CA, USA).

## Results

### Study Formulas’ Protein Fraction Evaluation

**Figure 1:**
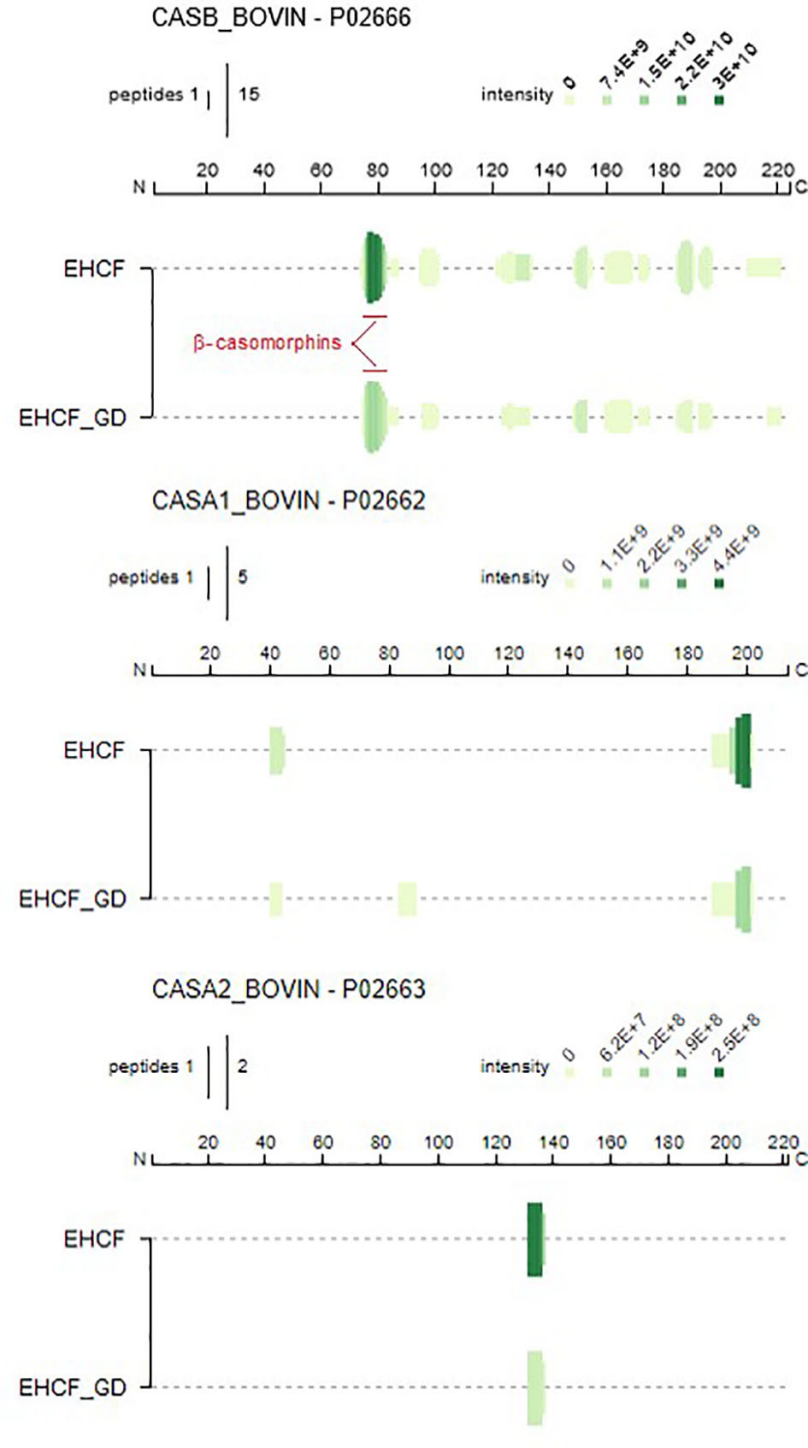
Schematic comparison (Peptigrams) of the peptide entries identified in EHCF and EHCF *in vitro* digested peptides. Maps have been visualized using the open source tool Peptigram (http://bioware.ucd.ie/peptigram/).

Potential batch-to-batch variability of the protein fraction for the study formulas was investigated. No relevant batch-to-batch protein fraction variations were observed for the five study formulas (**Figures S1A, B**). Protein fraction from EHCF and EHCF digests resulted in 61 and 40 casein-derived peptides, which primarily originated from *β*-casein and to a lesser extent from *α*_s1_-casein and *α*_s2_-casein, respectively (**Table S3**). The patterns of peptides in EHCF and EHCF digests were substantially unmodified among three different sample batches, as confirmed by comparative analysis of the chromatograms (**Figure S1A**). Apart from the 13 amino acid-long *α*_s1_-casein f (174–176), no peptide longer than 10 amino acid residues was detected in EHCF. The peptide maps of casein fragments in EHCF and EHCF digests were visualized using Peptigram plots, where the green shade intensity indicates the peptide amount (**Figure 1**). In **Figure S2**, we reported a detailed map of the *β*-casein-derived sequences, evidencing that peptide fragments originating from the *β*-casomorphin region are among the most abundant in EHCF, and for the greatest part they survived the simulated infant gastroduodenal digestion. However, as shown in **Figure S3**, we confirmed the absence of oligopeptides in AAF at detectable amount.

### Gut Barrier Integrity

Protein fraction from different study formulas did not affect intestinal epithelial permeability, as demonstrated by TEER measurement up to 72 h of incubation in Caco-2 cells (**Figure S4**).

**Figure 2:**
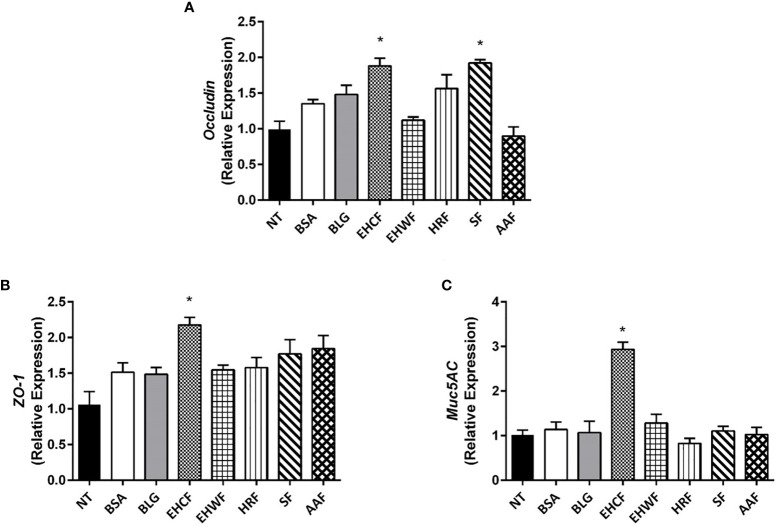
Modulation of gut barrier components (occludin, ZO-1 and Muc5AC) elicited by the study formulas-derived protein fraction in human enterocytes (Caco-2 cells). The 48 h incubation of Caco-2 cells with EHCF-derived protein fraction (25 µg/ml) stimulated the production of all three components of the gut barrier that were investigated in Caco-2 cells monolayer: Occludin **(A)**, Zonula occludens-1 (ZO-1) **(B)** and Muc5AC **(C)**. SF was able to stimulate the expression of occludin only **(A)**. The other three study formulas were unable to modulate the three gut barrier components. Data represent the means with SD of three independent experiments, each performed in triplicate. Data were analyzed using the paired t-test. *p < 0.05 *vs* untreated cells (NT). Muc5AC, mucin 5AC; EHCF, extensively hydrolyzed casein formula; EHWF, extensively hydrolyzed whey formula; HRF, hydrolyzed rice formula; SF, soy formula; AAF, amino acid-based formula.

Occludin expression was significantly up-regulated by protein fraction from EHCF and SF (**Figure 2A**), whereas only EHCF-derived protein fraction induced an increased expression of ZO-1 (**Figure 2B**). Similarly, only EHCF digests up-regulated the expression of Muc5AC in Caco-2 cells (**Figure 2C**).

Experiments performed with NCM460 cell line confirmed was what observed in Caco-2 cell line: the protein fraction from different study formula was unable to modulate TEER (**Figure S5A**). EHCF-derived protein fraction up-regulated occludin (**Figure S5B**), ZO-1 (**Figure S5C**), while SF was able to stimulate the expression of occludin only (**Figure S5B**).

### Epithelial Cell-Derived Danger Signal Mediators

**Figure 3:**
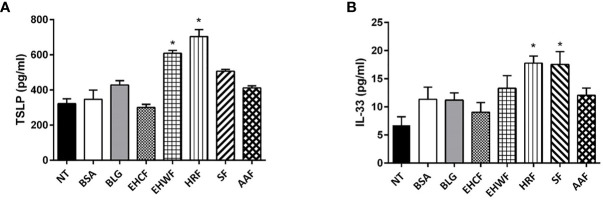
Modulation of the danger signal mediators thymic stromal lymphopoietin and interleukin 33 in human enterocytes (Caco-2 cells). The 48 h incubation of Caco-2 cells with EHRF-derived protein fraction (25 µg/ml) resulted in a stimulation of both biomarkers of gut epithelial cells’ danger signal mediators’ production: thymic stromal lymphopoietin (TSLP) **(A)** and interleukin (IL)-33 **(B)**. TSLP production also increased upon stimulation with 25 µg/ml EHWF and HRF **(A)**, whereas IL-33 production increased stimulating Caco-2 cells with 25 µg/ml SF and HRF **(B)**. EHCF and AAF-derived protein fractions were unable to modulate the two biomarkers. Data represent the means with SD of three independent experiments, each performed in triplicate. Data were analyzed using the paired t-test. *p < 0.05 *vs* untreated cells (NT). TSLP, thymic stromal lymphopoietin; IL-33, interleukin-33; EHCF, extensively hydrolyzed casein formula; EHWF, extensively hydrolyzed whey formula; HRF, hydrolyzed rice formula; SF, soy formula; AAF, amino acid-based formula.

Protein fraction derived from EHWF and HRF elicited an increase in TSLP production in Caco-2 cells (**Figure 3A**). IL-33 release was increased by protein fraction from HRF and SF (**Figure 3B**). No modulation of these two cytokines was observed exposing human enterocytes to the protein fraction from the other study formulas.

### Th1 and Th2 Cytokines Response in PBMCs From CMA Infants

**Figure 4:**
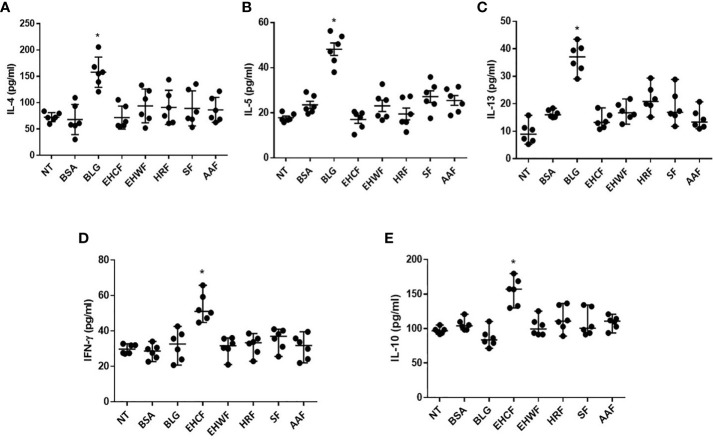
Modulation of Th2/Th1 cytokines production in peripheral mononuclear blood cells from infants affected by cow’s milk allergy. Exposing peripheral mononuclear blood cells (PBMCs) collected from six infants affected by IgE- mediated cow’s milk allergy for 5 days with 25 µg/ml *β*-lactoglobulin (BLG) resulted in a significant increase of all Th2 cytokine production: IL-4 **(A)**, IL-5 **(B)**, IL-13 **(C)**. The protein fractions derived from the study formulas were unable to increase the production of Th2 cytokines. Whereas, only EHCF-derived protein fraction was able to increase IL-10 **(D)** and IFN-*γ* **(E)** production. Each data point represents the single patient response. Horizontal bars represent the means with SD obtained within each group. Data were analyzed using the paired t-test. *p < 0.05 *vs* untreated cells (NT). BLG, β-lactoglobulin; EHCF, extensively hydrolyzed casein formula; EHWF, extensively hydrolyzed whey formula; HRF, hydrolyzed rice formula; SF, soy formula; AAF, amino acid-based formula.

All protein fractions from the different study formulas were unable to modulate IL-4, IL-5, and IL-13 production in PBMCs from IgE-mediated CMA infants (**Figures 4A–C**). Conversely, only EHCF-derived protein fraction was able to significantly up-regulate the IFN-*γ* and IL-10 production (**Figures 4D, E** **)**. IL-10 and IFN-*γ* data were further evaluated according to casein sensitization status of CMA patients, and no differences were observed comparing PBMCs’ response in patients with or without casein sensitization. No modulation in DNA methylation status of IFN-*γ* and IL-10 genes was observed for EHCF and for the other study formulas.

### Treg Activation in PBMCs From CMA Infants

**Figure 5:**
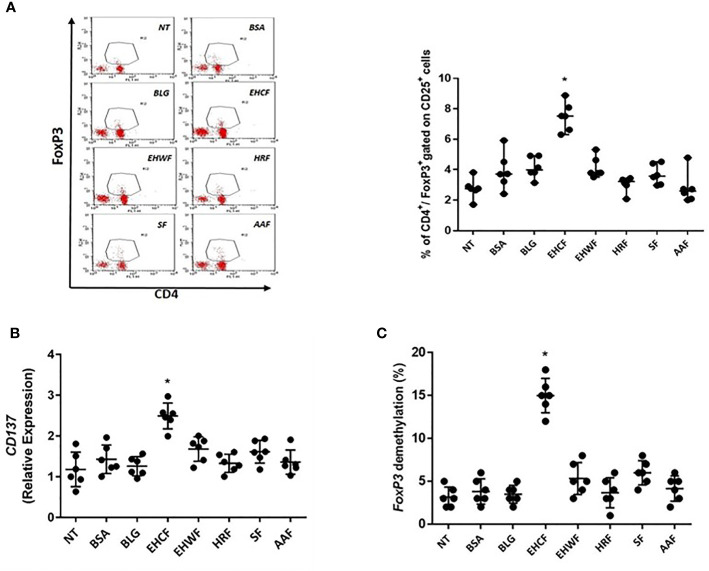
Regulatory T cells’ activation in peripheral mononuclear blood cells from infants affected by cow’s milk allergy. Exposing peripheral mononuclear blood cells (PBMCs) collected from six infants affected by IgE-mediated cow’s milk allergy for 5 days with 25 µg/ml EHCF-derived protein fraction resulted in a stimulation of CD4+/CD25+/FoxP3+ cells’ number **(A)**. An up-regulation of CD137 expression, a marker of Treg activation, was observed in PBMCs stimulated only EHCF-derived protein fraction **(B)**. The effect paralleled with an up-regulation of *FoxP3* demethylation rate in Treg-specific-demethylation-region (TSDR) **(C)**. All other study formula-derived protein fractions were unable to modulate regulatory T cells activation. Each data point represents the single patient response. Horizontal bars represent the means with SD obtained within each group. Data were analyzed using the paired t-test. *p < 0.05 vs untreated cells (NT). TSDR, Treg-specific-demethylation-region; EHCF, extensively hydrolyzed casein formula; EHWF, extensively hydrolyzed whey formula; HRF, hydrolyzed rice formula; SF, soy formula; AAF, amino acid-based formula.

Only EHCF-derived protein fraction was able to significantly increase CD4^+^/CD25^+/^FoxP3^+^ Treg number and up-regulated CD137 expression, a marker of activated Tregs (**Figures 5A, B** **)**. The effect paralleled with an up-regulation of *FoxP3* demethylation rate in TSDR (**Figure 5B**). Protein fractions from other study formulas were unable to modulate CD4^+^/CD25^+^/FoxP3^+^ Tregs, and its demethylation status in PBMCs from CMA infants (**Figures 5A–C**). In addition, we found that only EHCF-derived protein fraction significantly increased *NFAT*, *AP1*, and *Nf-Kb1* expression in Tregs from CMA children, further confirming a specific activation of Tregs through this intracellular pathway (**Figure S6** **)**.

## Discussion

Our findings demonstrate a different modulation on tolerogenic mechanisms elicited by the protein fraction derived from the formulas commonly used for CMA management.

The gut barrier-related non-immune mechanisms, such as epithelial permeability and mucus thickness, are considered relevant in preserving immune tolerance (32). Loss of gut barrier integrity increases antigen uptake and promotes Th2-type allergic response by activation of type 2 innate lymphoid cells (ILC2s), mast cells, basophils, and dendritic cells (DCs) (33). We found that EHCF- derived protein fraction could improve gut barrier integrity increasing occludin, ZO-1, and Muc5AC expression in human enterocytes. These data are well in line with previous observation reporting the up-regulation of Muc5AC expression after stimulation with casein hydrolysate in human enterocytes (34). Of note, we observed that also SF-derived protein fraction stimulated occludin expression. Similarly, it has been previously demonstrated that peptides derived from fermented soybean improved epithelial barrier function, enhancing occludin expression (35). The other study formulas (EHWF, HRF, and AAF) were unable to modulate these components of gut barrier.

Epithelium-derived cytokines, including TSLP and IL-33, have a pivotal role in the development of allergic response at gut barrier surface and have been linked to the pathogenesis of type 2 inflammatory diseases, including food allergy and asthma (33). We found that protein fraction derived from EHWF, HRF, and SF elicited an increase in TSLP and/or IL-33 production. In contrast, the protein fractions from EHCF and AAF were unable to modulate the expression of such cytokines.

To further investigate the immunomodulatory effect elicited by protein fraction from the different study formulas, Th1 and Th2 cytokine production was measured in PBMCs from IgE-mediated CMA infants. It has been already demonstrated that allergen specific stimulation induced an immune reactivity in PBMCs from IgE-mediated CMA infants (36). All protein fractions from the different study formulas were unable to elicit Th2 cytokine response. But, only EHCF-derived protein fraction significantly enhanced the production of the Th1 cytokine IFN-*γ* and of the key mediator of immune tolerance IL-10. Similarly, it has been demonstrated that casein hydrolysates could induce a significant increase of IL‐10 levels in rats (37), but the results were not confirmed by others (12).

Clinical trials demonstrated that EHCF could accelerate immune tolerance acquisition in CMA children if compared with other dietary strategies through, at least in part, an epigenetic modulation of *FoxP3* gene (14, 30). CD4+/CD25+/FoxP3+ cells are central in the maintenance of immune homeostasis and tolerance. It has been demonstrated that the enzymatic digest of milk caseins was able to induce immune tolerance in the mouse model (38). We found that only EHCF-derived protein fraction elicited a significant activation of CD4+/CD25+/FoxP3+ Tregs, through DNA demethylation of the *FoxP3* transcription factor. This mechanism could be related to the activation of the transcription factor complex NFAT/Nf-Kb/AP1 on T cells partially methylated in *FoxP3* promoter (39, 40). The other study formulas (EHWF, HRF, SF and AAF) were unable to modulate such mechanism.

Amino acid-based formulas, consisting of essential and non-essential amino acids, are currently recommended for infants who do not tolerate extensively hydrolyzed formulas or with multiple and/or extremely severe or life-threatening food allergies (41). Although AAF is considered the safer dietary strategy for severe CMA infants, experimental and clinical data suggest that this formula is unable to promote tolerogenic effects, substantially due to the absence of peptides (14, 41). Indeed, using nanoflow-HPLC-MS/MS and HPLC-UV approach, we confirmed the absence of oligopeptides in AAF at detectable amount. Lower lymphocyte count in small intestine lamina propria, decreased Peyer’s patches size, lower number of FoxP3+ CD4+ Treg cells, with higher level of specific IgE against dietary antigens and higher severity of allergic manifestations have been previously associated with the use of AAF (42). The results of the present study, showing absence of effects on gut barrier components, Th1/Th2 cytokine response and Tregs activation, are well in line with these previous findings.

A study limitation is due to the fact that we didn’t identify which specific EHCF-derived peptides or amino acid sequences were responsible for the tolerogenic effects. However, we performed a characterization of EHCF-derived peptides and we detected the presence of several peptides with a previously described immunomodulatory action. A relatively high number of peptides in the EHCF-derived protein fraction derived from the *β*-casomorphin domain of *β*-casein, which is considered a “strategic” sequence because it can release potentially potent bioactive peptides (43, 44). *β*-casomorphins and related peptides detected in EHCF may also prevent the uptake of luminal antigens, as they stimulate intestinal mucin production (45). Additional EHCF-derived peptides were *β*-casein f (134–138) (sequence: HLPLP), and *β*-casein f (177–183) (sequence: AVPYPQR) (43). The peptides from both these regions have been found to inhibit ACE and may act on the immune system by preventing the breakdown of bioactive bradykinin, which importantly contributes to immunoregulation by inducing maturation of DCs by driving Th1 oriented-response (46). Noteworthy, several EHCF-derived peptides shared the common amino acid motif PFP (Pro-Phe-Pro), deriving from three different positions within *β*-casein, namely 61–63, 110–112 and 204–206. The amino acid motif PFP has been previously indicated as the possible determinant of the immunoregulatory activity of *β*-casein regions (47, 48). Another limitation of the study could be related to the lack of identification of human cell receptors involved in the tolerogenic response. Cow’s milk protein hydrolysates binding on Toll like receptors (TLRs) have been demonstrated in epithelial cells (7), but more research is needed to evaluate the direct interaction with TLRs on immune cells. Lastly, we didn’t explore the effect of other components of commercially available formulas for CMA treatment potentially able to elicit tolerogenic effects, such as polyunsaturated fatty acids or vitamin D (49). It should be considered that these components occur at very similar concentration among different commercially available formulas. For these reasons, we focused only on the protein fractions, which largely differ among the commercially available formulas for CMA treatment. Thus, a strength of our study is that we investigated the effect deriving by the direct interaction of purified protein fractions with human cells. This could have reduced the bias of previous similar observation exploring the effects of whole infant formulas on human cells (12).

In conclusion, our results suggest a different regulatory action on immune and non-immune tolerogenic mechanisms elicited by protein fraction from different formulas for CMA management. In particular, we found that EHCF-derived protein fraction could activate several tolerogenic mechanisms through, at least in part, an epigenetic regulation of gene expression. These results could explain the beneficial effects observed on immune tolerance acquisition in CMA patients and on allergy prevention in children at risk for atopy (3–5, 14). The precise identification of EHCF peptides responsible for these effects, together with a better definition of the tolerogenic mechanisms elicited by such peptides, could provide pivotal information and could inspire the composition of next generation hypoallergenic formulas for the prevention and treatment of CMA.

## Data Availability Statement

The HPLC-MS/MS data are deposited in the ProteomeXchange Consortium (http://www.proteomexchange.org) via the PRIDE repository and can be accessed with identifier PXD023355 (50).

## Author Contributions

RBC designed, structured, and coordinated the experiments, and wrote and read the manuscript. LPa performed experiments, analyzed data, and wrote and read the manuscript. CB, GP, LPi, VC, and AS performed the experiments, analyzed data, and read the manuscript. RBC, LCa, LCo, and TC cared for the patients, analyzed data, and read the manuscript. All authors contributed to the article and approved the submitted version.

## Funding

This work was supported in part by a grant of the Italian Ministry of Health grant (PE-2011- 02348447) devoted to the Department of Translational Medical Science of the University “Federico II” of Naples.

## Conflict of Interest

The Department of Translational Medical Science received research grants from Nestlè, Vevey, Switzerland; Kraft Heinz Chicago, Illinois, USA; Danone, Paris, France; Mead Johnson, Evansville, IN, USA; and Novalac, Paris, France.

The authors declare that the research was conducted in the absence of any commercial or financial relationships that could be construed as a potential conflict of interest.
