# Supplementary material for: Tolerogenic Effect Elicited by Protein Fraction Derived From Different Formulas for Dietary Treatment of Cow’s Milk Allergy in Human Cells
Source: Front Immunol. 2021 Feb 12;11:604075. doi: 10.3389/fimmu.2020.604075 (PMC7928417; doi:10.3389/fimmu.2020.604075)
Supplement: Supplementary file 1 [file DataSheet_1.docx]

**Supplementary Figures**

**Tolerogenic effect elicited by protein fraction derived from different formulas for dietary treatment of cow’s milk allergy in human cells**

Lorella Paparo, Gianluca Picariello, Cristina Bruno, Laura Pisapia, Valentina Canale, Antonella Sarracino, Rita Nocerino, Laura Carucci, Linda Cosenza, Tommaso Cozzolino, Roberto Berni Canani

**(A)**


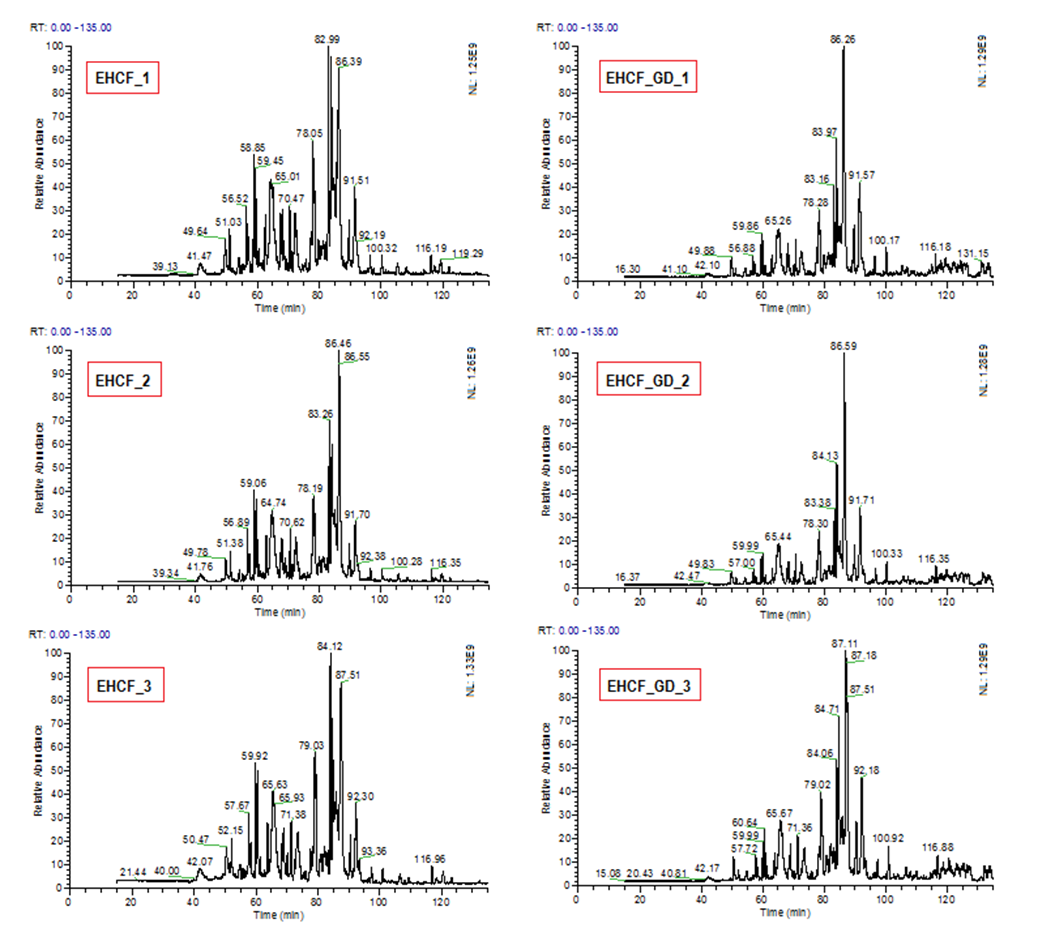


**(B)**


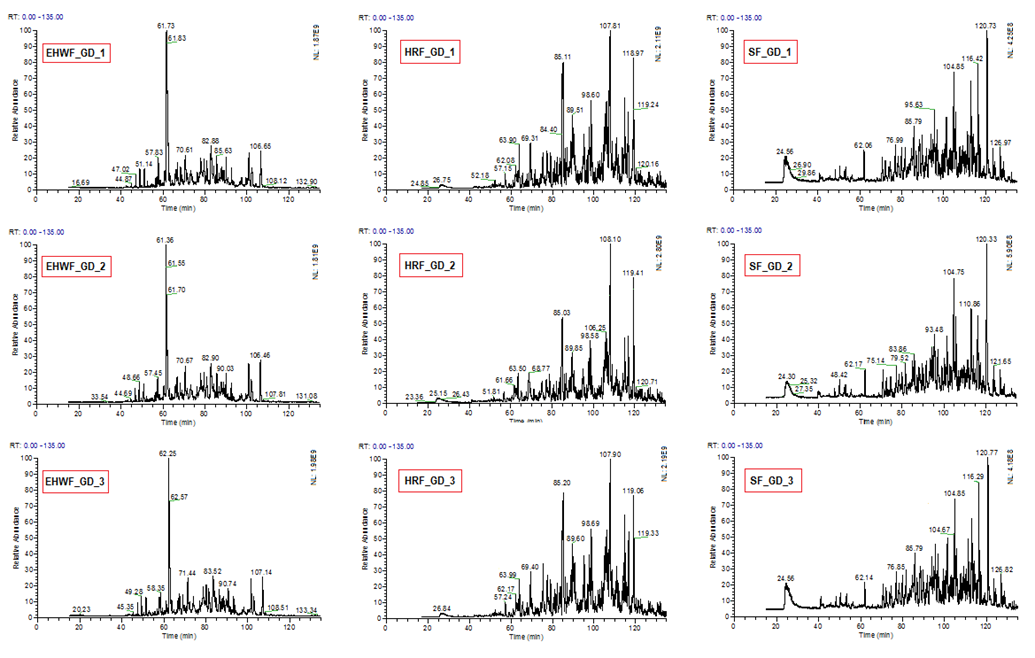


**Figure S1.**

**(A)** Total ion current (TIC) nanoflow-HPLC-MS/MS chromatograms of peptides purified from three batches of EHCF (left panels) and from the corresponding EHCF gastroduodenal digests (EHCF_GD, right panels). The strict similarity of samples purified from the three different batches supports the substantial equivalence of the extracts used in triplicate biological assays.

**(B)** Total ion current (TIC) nanoflow-HPLC-MS/MS chromatograms of three different preparations of in vitro gastroduodenal digests (infant model) from extensively hydrolyzed whey (EHWF), hydrolized rice (HRF) and soy-based (SF) formulas from three batches. The comparative analysis demonstrated very low inter-batches variability.


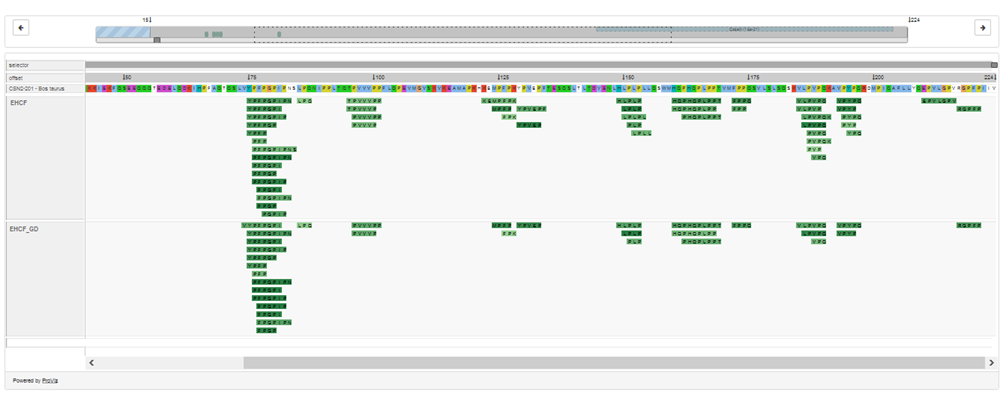


**Figure S2.** **Map of the β-casein-derived sequences.** Peptide fragments originated from the β-casomorphin region were among the most abundant in EHCF and in large part they survived the simulated digestion (peptides in EHCF_GD). The intensity of green colour indicates the peptides abundance.


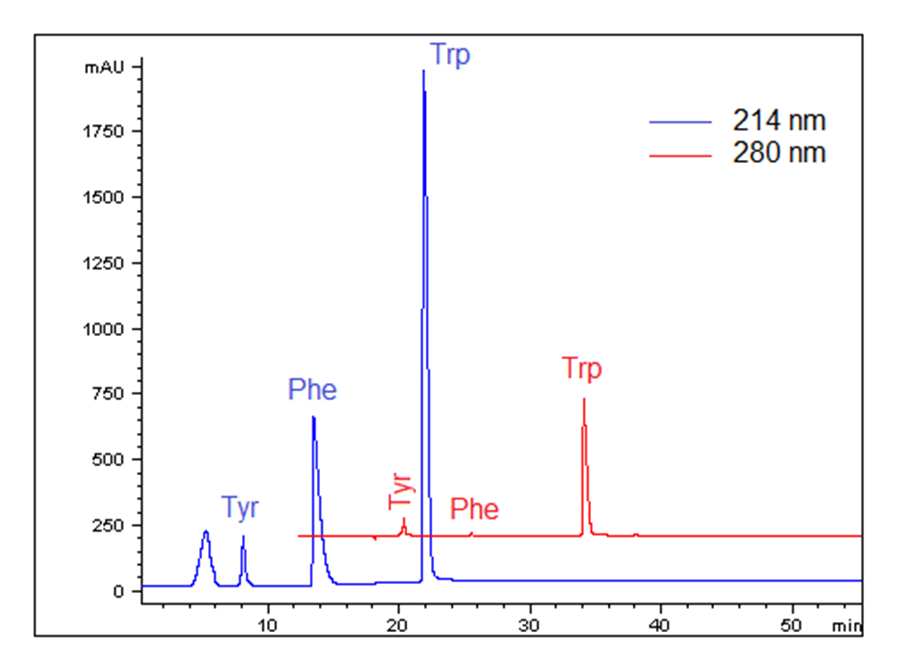


**Figure S3. HPLC-UV analysis of AAF**. Chromatograms were recorded at 214 and 280 nm, corresponding to the UV absorption bands of peptide bonds and aromatic rings. The analysis confirmed the absence of oligopeptides, also including di-/tri-peptides, at detectable amount.


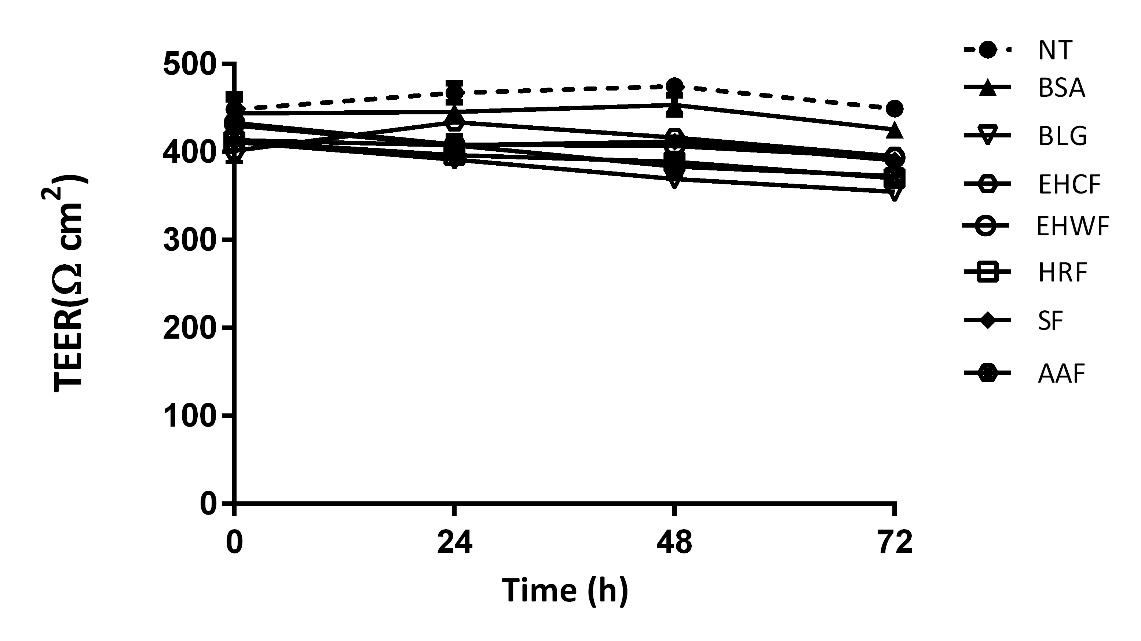


**Figure S4**. **Effect of protein fractions derived from study formulas on transepithelial electric resistance (TEER) as biomarker of intestinal epithelial permeability in Caco-2 cells.**

Protein fraction (25 µg/ml) from different study formulas did not affect intestinal epithelial permeability, as demonstrated by TEER measurement up to 72 hours of incubation. The TEER values were measured as follows: TEER = (measured resistance value−blank value) × single cell layer surface area (cm^2^). Data represent the means with SD of 3 independent experiments, each performed in triplicate.

*TEER, transepithelial electric resistance; BLG, β-lactoglobulin; EHCF, extensively hydrolyzed casein formula; EHWF, extensively hydrolyzed whey formula; HRF, hydrolyzed rice formula; SF, soy formula; AAF, amino acid-based formula*

*
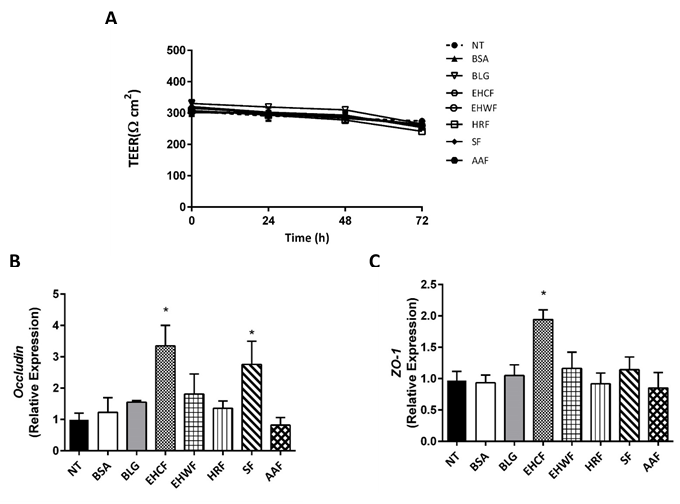
*

**Figure S5**. **Effect of protein fractions derived from study formulas on transepithelial electric resistance (TEER) and tight junction proteins (occludin and ZO- 1) as biomarkers of intestinal epithelial permeability in normal human colon epithelial cell line (NCM460).**

Protein fraction (25 µg/ml) from different study formulas did not affect intestinal epithelial permeability in NCM460 cell line, as demonstrated by TEER measurement up to 72 hours of incubation **(A)**. The TEER values were measured as follows: TEER = (measured resistance value−blank value) × single cell layer surface area (cm2). The 48h incubation with EHCF-derived protein fraction (25 µg/ml) stimulated Occludin **(B)** and Zonula occludens 1 (ZO-1) **(C)** expression. SF was able to stimulate the expression of occludin only **(B)**. The other three study formulas were unable to modulate the tight junction proteins expression.Data represent the means with SD of 3 independent experiments, each performed in triplicate. Data were analyzed using the paired t-test. **p*<0.05 *vs* untreated cells (NT).

*TEER, transepithelial electric resistance; EHCF, extensively hydrolyzed casein formula; EHWF, extensively hydrolyzed whey formula; HRF, hydrolyzed rice formula; SF, soy formula; AAF, amino acid-based formula.*


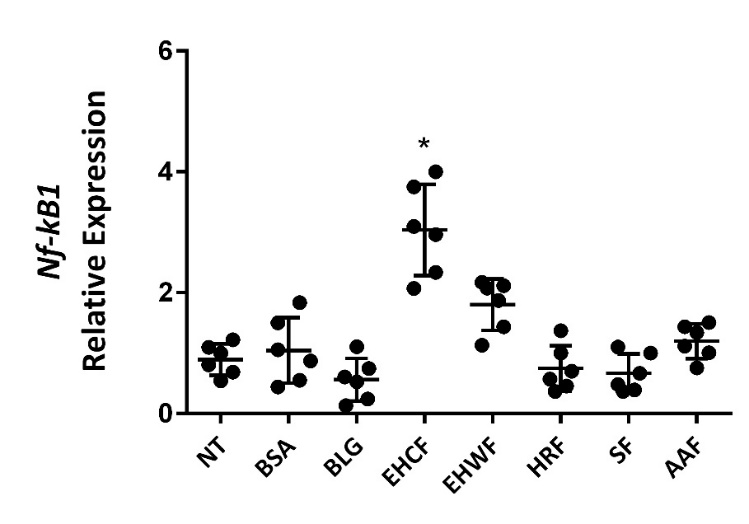

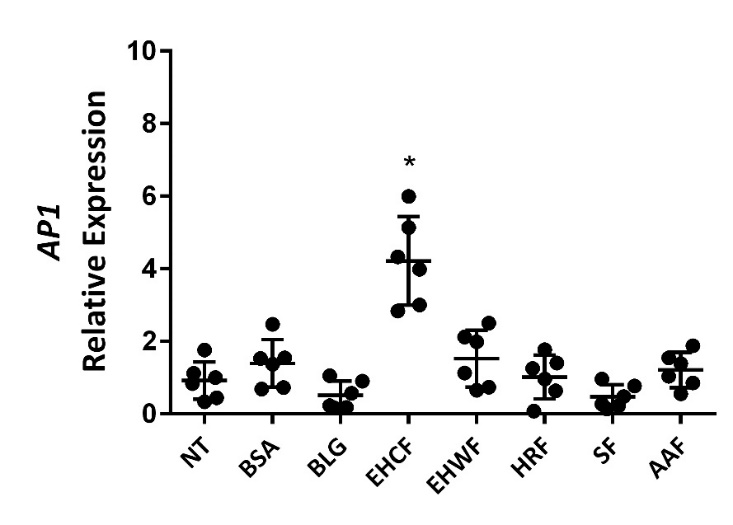

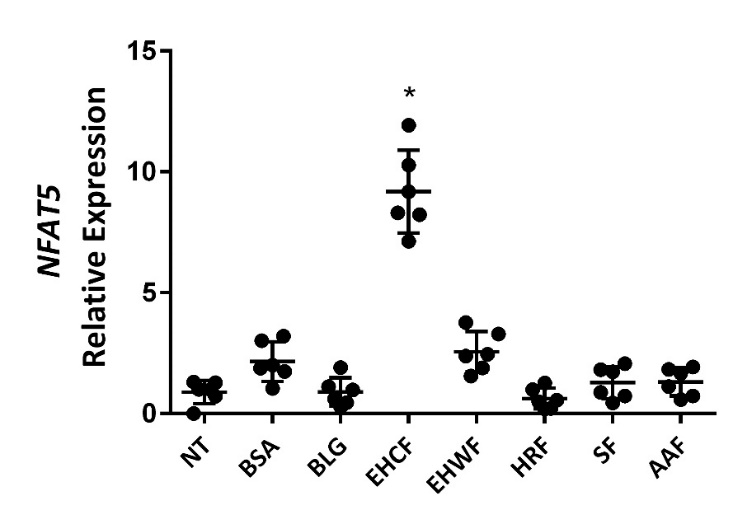


**A**

**B**

**C**

**Figure S6**. **Effect of study formulas-derived protein fraction on the transcription factor complex *NFAT5, AP1* and *Nf-kB1* expression.**

Exposing peripheral mononuclear blood cells (PBMCs) collected from six infants affected by IgE- mediated cow’s milk allergy for 5 days with 25 µg/ml EHCF-derived protein fraction resulted in increase of the transcription factor complex *NFAT5* **(A)**, *AP1* **(B)**, *Nf-kB1* **(C)** expression.

Each data point represents the single patient response. Horizontal bars represent the means with SD obtained within each group. Data were analyzed using the paired t-test. **p*<0.05 *vs* untreated cells (NT).

*BLG, β-lactoglobulin; EHCF, extensively hydrolyzed casein formula; EHWF, extensively hydrolyzed whey formula; HRF, hydrolyzed rice formula; SF, soy formula; AAF, amino acid-based formula.*
