# Supplementary figures and images for: Tolerogenic Effect Elicited by Protein Fraction Derived From Different Formulas for Dietary Treatment of Cow’s Milk Allergy in Human Cells
Source: Front Immunol. 2021 Feb 12;11:604075. doi: 10.3389/fimmu.2020.604075 (PMC7928417; doi:10.3389/fimmu.2020.604075)

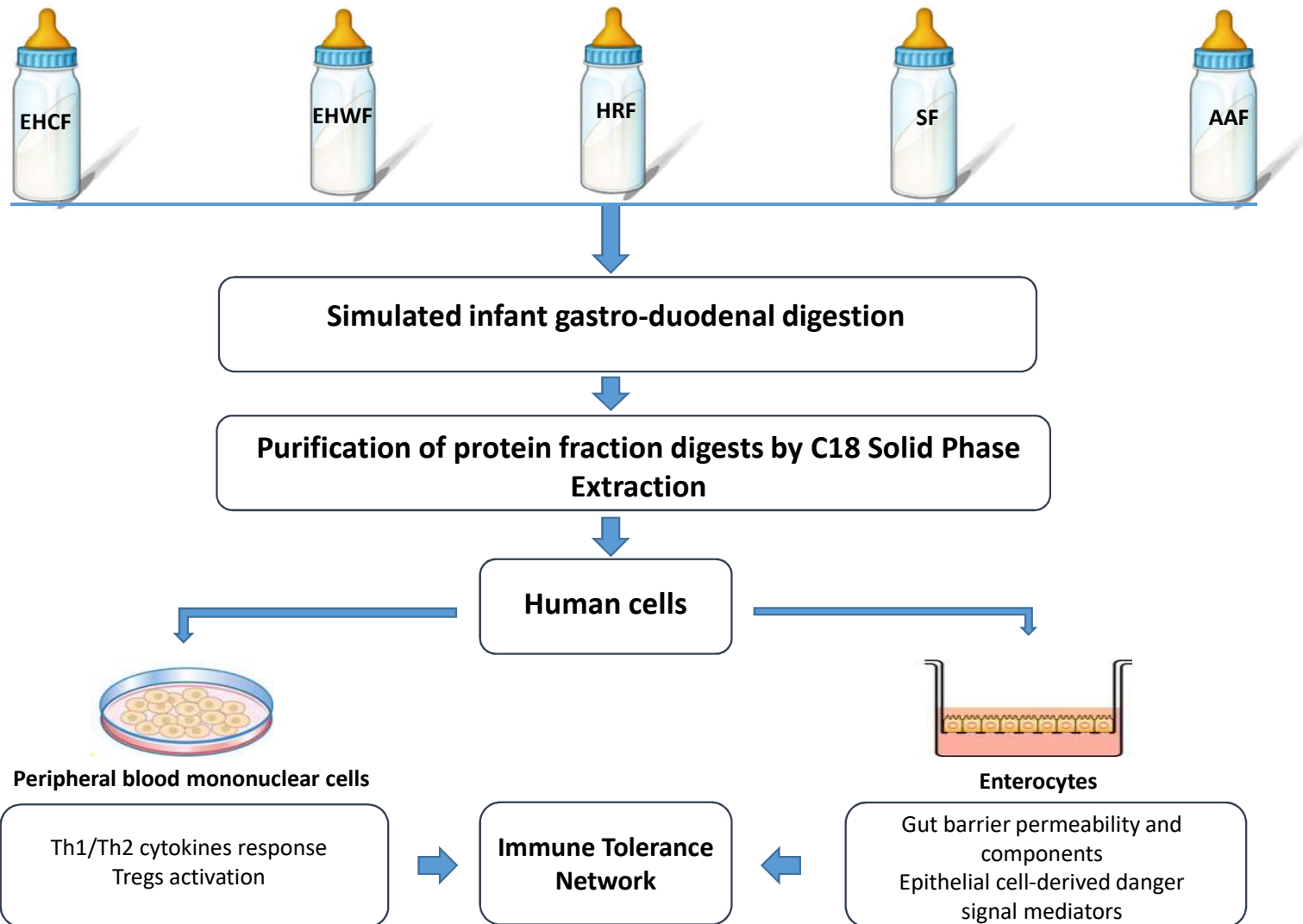

Supplement: Supplementary file 2 [file DataSheet_1.pdf]
